# RP-HPLC-Based Flavonoid Profiling Accompanied with Multivariate Analysis: An Efficient Approach for Quality Assessment of *Houttuynia cordata* Thunb Leaves and Their Commercial Products

**DOI:** 10.3390/molecules28176378

**Published:** 2023-08-31

**Authors:** Minh Hien Nguyen, Dieu Ly Ha, Binh Minh Do, Ngoc Trong Nghia Chau, Thi Huong Tran, Nguyen Thien Han Le, Minh Tri Le

**Affiliations:** 1 School of Medicine, Vietnam National University Ho Chi Minh City, Quarter 6, Linh Trung Ward, Thu Duc District, Ho Chi Minh City 700000, Vietnam; hdly@medvnu.edu.vn (D.L.H.); dbminh.d2017@medvnu.edu.vn (B.M.D.); cntnghia.d2017@medvnu.edu.vn (N.T.N.C.); tthuong.duoc2016@medvnu.edu.vn (T.H.T.); lnthan.d2019@medvnu.edu.vn (N.T.H.L.); 2 Vietnam National University Ho Chi Minh City, Quarter 6, Linh Trung Ward, Thu Duc District, Ho Chi Minh City 700000, Vietnam; 3 Faculty of Pharmacy, University of Medicine and Pharmacy of Ho Chi Minh City, Dinh Tien Hoang Street, Ben Nghe Ward, 1 District, Ho Chi Minh City 700000, Vietnam

**Keywords:** *Houttuynia cordata*, HPLC-based flavonoid profiling, flavonoids, hierarchical cluster analysis, principal component analysis

## Abstract

Chemical profiling for quality monitoring and evaluation of medicinal plants is gaining attention. This study aims to develop an HPLC method followed by multivariate analysis to obtain HPLC profiles of five specific flavonoids, including rutin (1), hyperin (2), isoquercitrin (3), quercitrin (4), and quercetin (5) from *Houttuynia cordata* leaves and powder products and assess the quality of *H. cordata* samples. Eventually, we successfully established HPLC-based flavonoid profiles and quantified the contents of 32 *H. cordata* fresh leave samples and four powder products. The study also quantified the contents of those five essential flavonoids using an optimized RP-HPLC method. Peak areas of samples were then investigated with principal component analysis (PCA) and hierarchical cluster analysis (HCA) to evaluate the similarity and variance. Principal components in PCA strongly influenced by hyperin and quercetin showed that the samples were clustered into subgroups, demonstrating *H. cordata* samples’ quality. The results of HCA showed the similarity and divided the samples into seven subgroups. In conclusion, we have successfully developed a practical methodology that combined the HPLC-based flavonoid profiling and multivariate analysis for the quantification and quality control of *H. cordata* samples from fresh leaves and powder products. For further studies, we will consider various environmental factors, including climate and soil factors, to investigate their effects on the flavonoid contents of *H. cordata*.

## 1. Introduction

The chemical profile describes the representation of chemical information of medicinal plants into a chromatogram or spectrogram as meaningful evidence for the rapid identification of herbs. It has been an attractive option in assessing the quality of medicinal herbs with various benefits, such as detecting counterfeit, adulterated, or poor-quality herbal plants through their principal natural compound groups. Currently, the chemical profiles of medicinal plants have been recommended as a technique for quality estimation of medicinal plants by the US Food and Drug Administration (FDA) [1], the European Medicines Agency (EMA) [2], the World Health Organization (WHO) [3], and many monographs of Chinese Pharmacopoeia [4] and Vietnamese Pharmacopoeia [5].

Among numerous analytical techniques for obtaining medicinal herb chemical profiles, including thin-layer chromatography (TLC) [6], high-performance thin-layer chromatography (HPTLC) [7], Fourier-transform infrared spectroscopy (FTIR) [8], high-performance liquid chromatography (HPLC) [9], and gas chromatography (GC) [10], HPLC is widely recognized as a powerful and conventional technique due to its high sensitivity, specificity, low detection limit, high accuracy, and precision [11]. Furthermore, HPLC provides qualitative and quantitative information about the sample more efficiently than other analytical techniques. However, an efficient and intuitive method to integrate and describe the data is required because HPLC generates a large amount of data. Multivariate statistical analysis approaches can be deployed for reducing data and extracting relevant information. In particular, principal component analysis (PCA) and hierarchical clustering analysis (HCA) are most commonly used in HPLC chromatography analysis [12,13,14]. PCA reduces raw data size and turns it into smaller data size without losing information in the original data. Therefore, fewer principal components (PCs) are needed to explain the correlation between abundant variables. Meanwhile, HCA aids in grouping data clusters, thereby visualizing the relationship of the analytic samples. HPLC-based profiles combined with PCA and HCA could effectively identify the classification and quality control of herbal medicines [9,15,16].

*Houttuynia cordata* (*H. cordata*, HC) is a popular vegetable grown throughout Vietnam. *H. cordata* belongs to the Saururaceae family and was originally described as the only species in the Houttuynia genus. In 2001, Zhu et al. discovered and characterized a second species, *Houttuynia emeiensis*, found in China; however, its validity is still unestablished [17]. Due to their heat-clearing, detoxifying, diuretic, and antiseptic properties, *H. cordata* has long been used to treat many diseases, such as constipation, hemorrhoids, furuncle, and urinary retention [18,19]. Many pharmacological effects of *H. cordata* have been demonstrated, including antioxidant [20], anti-inflammatory [21], and antibacterial properties [22]. These potentials are primarily due to flavonoid chemicals found in *H. cordata*, namely, rutin, quercetin, quercitrin, hyperin, and isoquercitrin [23,24]. Flavonoids are one of the main bioactive compounds and are of particular interest since they define the quality and capacity of *H. cordata*. Although the uses of *H. cordata* are undisputed, numerous studies in recent years have revealed many important newly discovered biological properties. Most studies on the flavonoid composition of *H. cordata* preferred to use the leaves components as the analysis sample because the highest content of total flavonoids was found in leaves, followed by roots and stems, with substantial differences [25]. The presence of quercitrin, quercetin, and hyperoside in *H. cordata* leaves was demonstrated to have potent ferric-reducing antioxidant power and DPPH radical-scavenging activity in vitro by lowering CCl_4_-induced oxidative stress in mouse liver [26]. Flavonoids of *H. cordata* were proposed to improve H1N1-induced acute lung injury in mice through inhibition of influenzal neuraminidase activity and TLR signaling, in which hyperin and quercitrin played the main roles in the therapeutic effect [27]. On the other hand, a flavonoid hyperoside-enriched fraction from *H. cordata* leaves was proposed to be UVB-induced photoprotective or anti-skin aging agent via the MAPK signaling pathway [28]. This clarified the usages of *H. cordata* in the pharmaceutical cosmetic industry.

Because of the potential and promising applications of *H. cordata*, it is necessary to develop an effective classification and quality assessment method to evaluate the flavonoid content in *H. cordata* across a variety of geographical locations in Vietnam and the commercial products after processing. The present study aims to develop an HPLC method to obtain chromatographic profiles of specific flavonoids of *H. cordata* leaves collected from different sites in Vietnam and *H. cordata* leave powder products available in the market. Simultaneously, this research quantified these five main flavonoids in *H. cordata* leaves and applied hierarchical cluster analysis (HCA) and principal component analysis (PCA) to evaluate the similarity and variance between *H. cordata* samples. It is essential to highlight the high-quality *H. cordata* growing regions and the quality of commercial products.

## 2. Results

### 2.1. Optimization of RP-HPLC Conditions for Flavonoid Analysis

Table 1 displays the various RP-HPLC conditions used to investigate the optimal method for the *H. cordata* leaves analysis, represented by each experiment’s retention time, resolution, tailing factor, and the number of theoretical plates. Firstly, we used the Phenomenex column (250 mm × 4.6 mm, 5 µm) to examine the ideal mobile phase. With acetonitrile as solvent B and formic acid in water at 0.1% (A), the chromatograms demonstrated more excellent resolution, tailing factor, and the number of theoretical plates of the analytical peaks than acetic acid at 0.2% (A). As a result, formic acid 0.1% (A) and acetonitrile (B) were utilized as mobile phases for further investigation with a 150 mm column at two column temperatures of 25 °C and 40 °C and a lower flow rate (0.6 mL min^−1^). At 25 °C, the two closest peaks, hyperin and isoquercitrin, have the highest resolutions (5.221 and 0.909, respectively). The tailing factor of peaks range from 0.8 to 1.2, and the number of theoretical plates greater than 5000 indicate that the peaks have good symmetrical shapes. By increasing the column temperature to 40 °C, the parameters are not better than at 25 °C. Thus, a 150 mm column at 25 °C was selected as the optimal condition for HPLC-based profiling and quantifying five specific flavonoids from *H. cordata* leaves.

**Figure 1:**
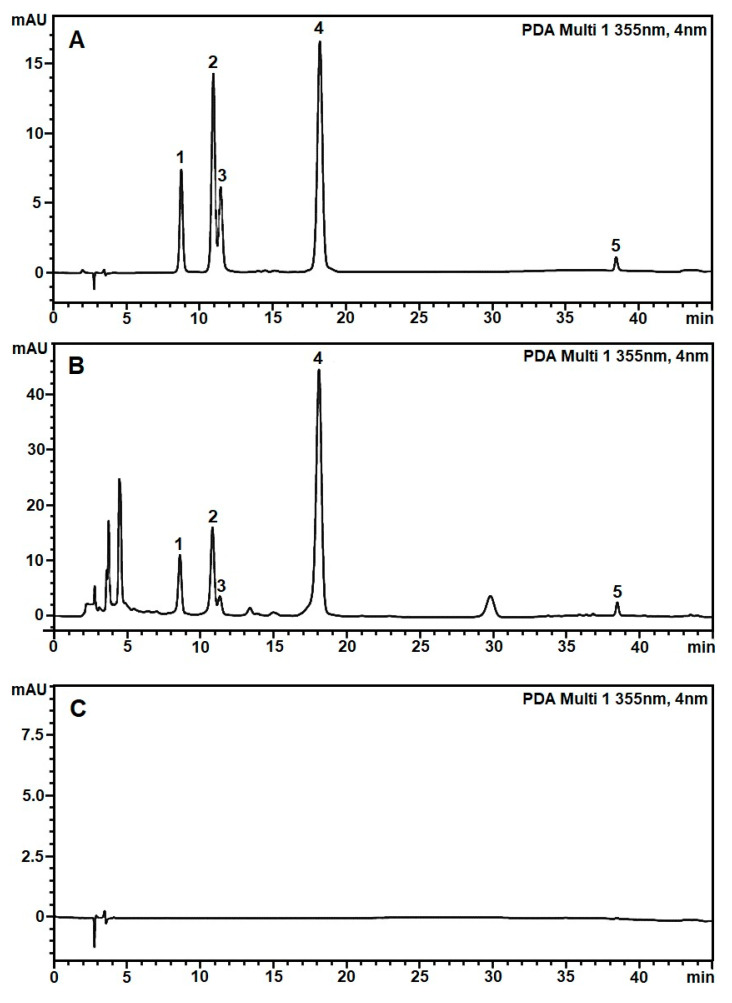
The typical RP-HPLC profile of five standard flavonoids (**A**), flavonoids in the leave extract of *H. cordata* (**B**), and solvent mobile phase baseline (**C**) were achieved under the optimized RP-HPLC methods with elution program C, Phenomenex column 150 mm × 4.6 mm × 5 μm at a flow rate 0.6 mL/min, an injection volume of 2 μL, and a column temperature at 25 °C. The peaks marked with 1 to 5 were of rutin (8.74 min), hyperin (10.93 min), isoquercitrin (11.43 min), quercitrin (18.22 min), and quercetin (38.46 min), respectively.

The chromatogram denoted the target flavonoid peaks with the best separation under the optimal HPLC conditions using Phenomenex C18 reversed-phase column (150 mm × 4.6 mm, 5 μm), and the mobile phase consisted of formic acid 0.1% in water (A) and acetonitrile (B) with a gradient program of 20% B in 0–5 min, 20–21% B in 5–25 min, and 21–50% B in 25–45 min, a flow rate of 0.6 mL min^−1^, an injection volume of 2 μL, and a column temperature kept at 25 °C. The wavelength of detection was 355 nm for acquiring chromatograms. The total analysis duration was 45 min. The typical optimized HPLC chromatograms are shown in Figure 1.

### 2.2. Validation of Optimized RP-HPLC Method

#### 2.2.1. Specificity

**Figure 2:**
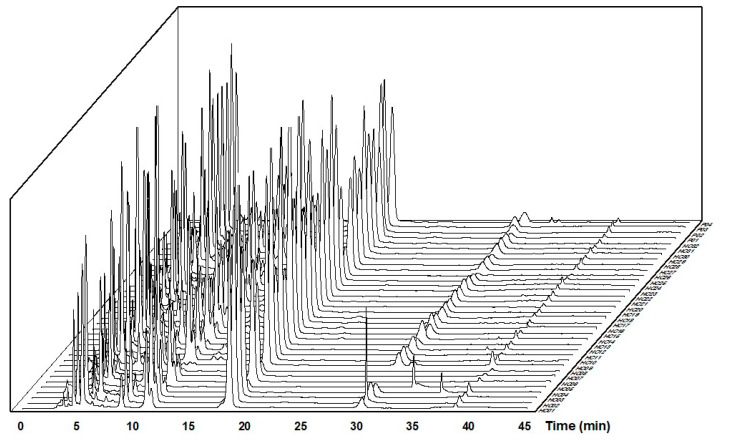
HPLC-based flavonoid profiles from 36 samples of *Houttuynia cordata* leaves are designated HC01-HC32 and P01-P04 on the z-axis.

The specificity of the method was validated by comparing the chromatograms of the mobile phase blank, the standard sample, and the *H. cordata* leaves extract sample using the optimized HPLC conditions in Section 2.1. Figure 2 shows the retention times of five specific flavonoids, including rutin, hyperin, isoquercitrin, quercitrin, and quercetin in the extracts, which were monitored at 8.74, 10.93, 11.43, 18.22, and 38.46 min, respectively. The chromatograms of the blank showed no analytes that interfered with absorption at 355 nm. The specificity ensured that the method was suitable for the qualitative and quantitative analysis of five flavonoids in *H. cordata* leaves and powder products.

#### 2.2.2. Precision, Repeatability, Intermediate Precision, and Stability

The HC27 leaves sample was analyzed six times to assess the precision. Stability was evaluated by analyzing samples of HC27 at different time points (0, 3, 6, 9, and 12 h). The *H. cordata* sample was analyzed five times to evaluate the repeatability. %RSD for peak area of five flavonoids, including rutin, hyperin, isoquercitrin, quercitrin, and quercetin, was less than 2% for all, reflecting the method’s precision. The *H. cordata* leaves test sample was determined within 12 h. The results are shown in Table 2.

#### 2.2.3. Accuracy

Three concentrations (80%, 100%, and 120%) of the standards were added to the HC27 sample to evaluate the accuracy of the quantitative method. The results are presented in Table 3. The recoveries of rutin, hyperin, isoquercitrin, quercitrin, and quercetin were all in the 98–102% range, indicating the method’s accuracy.

#### 2.2.4. Linearity, Range, Limit of Detection, and Limit of Quantification

Five calibration curves all had good linearity with R^2^ > 0.9999 and the quantification range was relatively wide. The limit of detection and quantification were also calculated based on the parameters’ standard deviation and slope of the calibration curve. The results are shown in Table 4.

### 2.3. HPLC-Based Flavonoid Profiles

HPLC-based flavonoid profiles of 36 samples of *H. cordata* leaves and four samples of powder products processed with Origin 2021 software were presented in Figure 2.

Figure 2 shows the corresponding chromatograms at the standard peaks, including the five specific flavonoids: rutin, hyperin, isoquercitrin, quercitrin, and quercetin. Chromatograms of samples HC04 and HC05 have strange peaks at 36.15 min and 33.24 min, respectively. The chromatogram of sample HC09 has differences in the retention time of rutin, hyperin, and isoquercitrin peaks. In sample HC05, the peak of quercetin was challenging to identify on the chromatogram due to low concentration.

### 2.4. Quantification Results of 36 H. cordata Leaves Samples

Five flavonoids, including rutin, hyperin, isoquercitrin, quercitrin, and quercetin, were quantitatively determined using substitutive standard substances. Table 5 describes the calibration curves for the quantification of each flavonoid. In the *H. cordata* samples, the content of quercitrin was the most (0.25–13.13 mg/g), followed by hyperin (0.02–2.22 mg/g), rutin (0.04–1.64 mg/g), isoquercitrin (0.02–0.98 mg/g), and quercetin (0.006–0.12 mg/g). In general, samples of HC10 and HC14 (Tien Giang and Quang Tri province, respectively) have high flavonoid content.

It was evident that quercitrin was one of the most abundant components in *H. cor-data*. It deserves to be a reference substance and index for quality assessment and control of *H. cordata*. Meanwhile, the results in Table 5 illustrated remarkable differences in the contents of the five marker substances from different regions, which could be attributed to the variations in genetics, plant origins, environmental factors, drying process, and storage conditions.

### 2.5. Principal Component Analysis (PCA)

The five flavonoid peak areas of 36 samples of *H. cordata* leaves were used as variables in the PCA. The PCs analysis results are presented in Table 6. PC1 and PC2 had the highest eigenvalues (3.47 and 0.96), explaining most of the total variance of the data, and they described 69.41% and 19.56% of the cumulative variance, respectively. PC3, PC4, and PC5 had little interaction with the variable.

**Figure 3:**
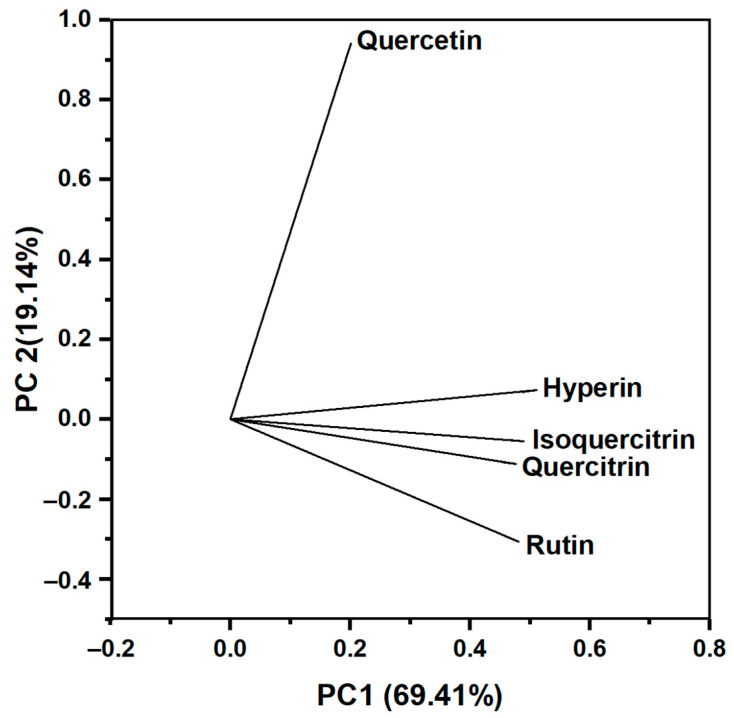
The loading plot illustrates the influence of flavonoids on the principal components.

A loading plot (Figure 3) is provided to examine the degree of influence of each flavonoid on the principal components. The closer the loadings are to ±1, the stronger the variable affects the component. Hyperin, isoquercitrin, rutin, and quercitrin vectors have significant positive loadings toward PC1. Hyperin, isoquercitrin, rutin, and quercitrin vectors have significant positive loadings toward PC1 (Table S3). Moreover, the vectors of hyperin, isoquercitrin, rutin, and quercitrin were close to one another, forming small angles, showing that they were positively correlated. Meanwhile, quercetin significantly affects PC2 with a strong coefficient (0.94097), and the quercetin vector formed an angle close to 90° with the other four vectors, showing that quercetin was almost uncorrelated with other flavonoids. Among the flavonoids in the PCA, hyperin and quercetin have the most contribution toward PCs with coefficients of 0.5115 (PC1) and 0.94097 (PC2), respectively. Therefore, we used hyperin and quercetin as principal components to analyze the relationship between samples.

**Figure 4:**
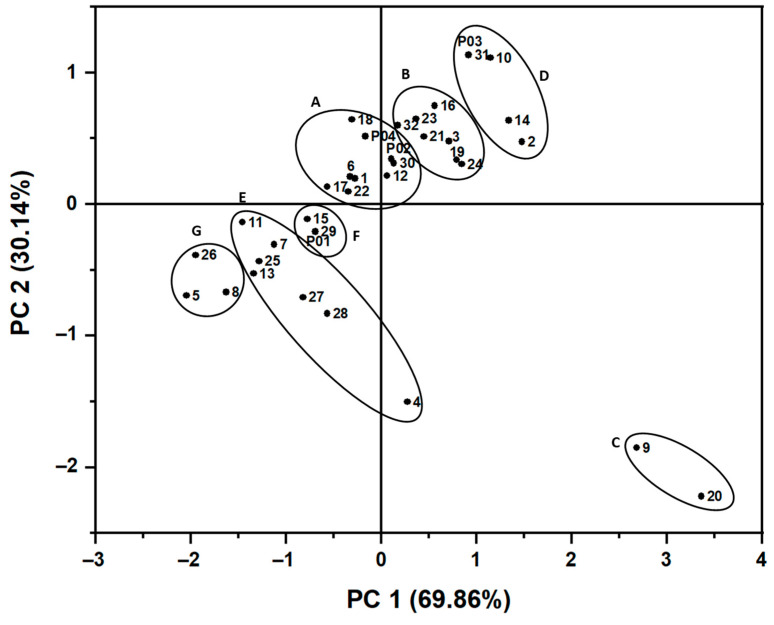
The score plot presents the distribution of *H. cordata* leaves samples on the same plane as well as the correlation between them and the first and second principal components. Based on HCA results, 36 samples (1–32 and P01–P04) are divided into seven groups labeled A–G.

The score plot represents the distribution of *H. cordata* leaves samples in the same plane (Figure 4). Quadrant I indicates the samples having a high content of hyperin, while quadrant II consists of the samples with an average hyperin amount. Quadrant III shows the cluster of samples with low concentrations of hyperin and quercetin. The group of samples with abnormally high quercetin content is in quadrant IV with HC09 and HC20 (0.1126 ± 0.0001 and 0.1265 ± 0.0012 mg/g extract, respectively). HC04 sample located in quadrant IV has a high concentration of quercetin, but it lies closer to the cluster in quadrant III due to the low amount of hyperin in this sample.

Moreover, the clusters of four commercial products labeled P01-P04 also showed that these products are different in flavonoid content from other samples. Three of *H. cordata*’s commercial products are in quadrant I (P02, P03) and quadrant II (P04), whereas P01 is located in quadrant III, meaning it has low hyperin and quercetin contents. Besides the culturing and harvesting conditions, the score plot may also indicate different drying methods. The freeze-drying method achieved P02, P03, and P04, while P01 was processed using the hot air-drying method.

### 2.6. Hierarchical Cluster Analysis (HCA)

**Figure 5:**
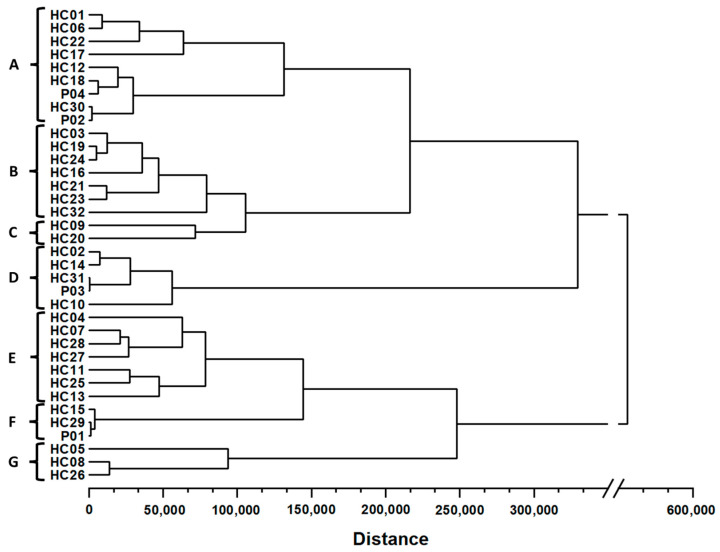
Hierarchical cluster analysis of 32 *H. cordata* leaves samples and four powder products. All samples were classified into nine clusters labeled A–G.

The similarity analysis of the *H. cordata* samples was performed based on the content of the five flavonoids of 36 samples, and the results are shown in Table S5. Within the same squared Euclidean distance, the HCA shows that there are seven subgroups from A to G (Figure 5) similar to the clustering results of PCA score plot.

In subgroup A (HC01, HC06, HC22, HC17, HC12, HC30, P04, and P02), most samples were collected from the southern provinces, but two were from the central provinces (HC17 and HC12). In contrast, most samples in subgroup B (HC03, HC19, HC24, HC16, HC21, HC23, and HC32) were collected mainly in provinces in the central region, excluding HC24 in Binh Thuan (southeast region) and HC32 in the north of Vietnam. Meanwhile, the samples of subgroup D (HC02, HC19, HC24, P04, and HC10) all had high contents of flavonoids, but they were collected from three different regions. The same pattern can be observed for subgroup E (HC04, HC07, HC11, HC13, HC25, HC27, and HC28). With high concentrations of quercetin, samples HC09 and HC20 formed a separate subgroup C matching the PCA score plot. Subgroups E, F, and G had a greater distance than subgroups A, B, C, and D and were separated due to low concentrations of hyperin and quercetin. Generally, the HCA results strongly support and validate the qualitative analysis of the samples conducted using PCA.

**Figure 6:**
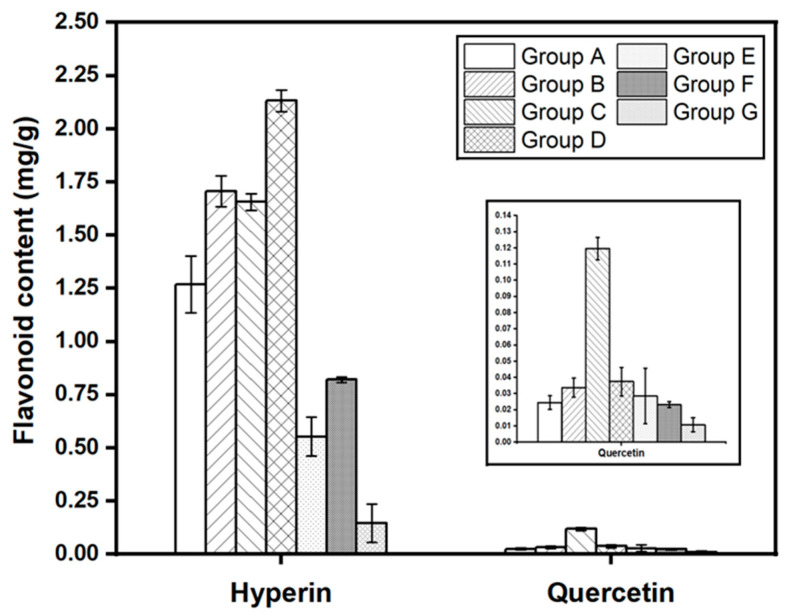
Comparison of hyperin and quercetin contents in *H. cordata* samples among seven groups.

Figure 6 demonstrates the differences between hyperin and quercetin contents divided into seven groups from multivariate analysis results. In terms of hyperin, there was a significant difference in the content of each group, excluding group B and group C, and have similar amounts of hyperin, 1.70697 ± 0.07343 and 1.65627 ± 0.03986, respectively (Table S4). However, the quercetin concentration of group C is 3.5 times higher than group B, leading to the clustering of *H. cordata* samples.

## 3. Discussion

Our studies successfully developed the RP-HPLC method for qualitative and quantitative analysis of five specific flavonoids in *H. cordata* leaves growing in Vietnam. Yang et al. (2014) studied the HPLC-based flavonoid profiling of 35 *H. cordata* samples grown in different regions of Guizhou province in China [24]. They determined that the rutin, isoquercitrin, quercitrin, and quercetin contents were 0.03–1.7 mg/mg, 0.01–1.19 mg/g, 0.02–5.50 mg/g, and 0.01–0.18 mg/g, respectively. Notably, the quercitrin content of Vietnamese *H. cordata* was 2.4 times higher than Chinese *H. cordata*. The concentrations of rutin, isoquercitrin, and quercetin were approximately the same as those found in *H. cordata* samples of this study. Wu et al. (2009) evaluated the flavonoid content in 22 *H. cordata* samples grown in different regions of China, in which the average hyperin, quercitrin, and quercetin content were 4.313 mg/g, 5.738 mg/ g, and 0.096 mg/g, respectively [29]. Quantitative results showed that the HC14 sample had a quercitrin content 2.3 times higher than the average quercitrin content of the Chinese *H. cordata* samples.

We performed a principal component analysis and hierarchical cluster analysis to evaluate the similarities and distinctions among samples based on the HPLC profiles. The purpose of using PCA is to reduce the dimensionality of the datasets and enhance data interpretability by preserving essential information [30]. When using five flavonoids to analyze, we found that clustering in the score plot did not clearly show the difference in flavonoid components among the samples (Figure S1). Meanwhile, when we selected hyperin and quercetin, the two flavonoids that most affect PCs, the score plot shows the clustering of the samples match with the HCA results. Therefore, the use of PCA based on two flavonoids contents, hyperin and quercetin, can demonstrate quality of *H. cordata*.

Previously, Ling-Shang et al. (2009) [29] have quantified the content of three flavonoids, including hyperin, quercitrin, and quercetin, in 23 *H. cordata* samples from different regions in China. Similar to our study, the results reveal that geographic factors did not significantly correlate to the flavonoid contents in *H. cordata*. Furthermore, Ling-Shang et al. evaluated the correlation between the content of those three flavonoids and plant morphology in these samples. On the other hand, our study used extracts from fresh leaves and powdered samples of *H. cordata* to quantify five major flavonoids: rutin, hyperin, quercitrin, isoquercitrin, and quercetin using RP-HPLC. Then, PCA was used as a preprocessing step before clustering to reduce the dimension from five flavonoids to two flavonoids (hyperin and quercetin). Eventually, this enabled us to explain almost the variances of the samples in HCA and PCA results. In our research, the herbal quality of *H. cordata* was determined based on quantitative results accompanied by multivariate analysis, not based on plant morphology, which is suitable for evaluating the quality of *H. cordata* powder products on the market. In the study of Qi et al. (2022) [25], they utilized the headspace solid-phase micro-extraction technique (HS-SPME-GC-MS), followed by orthogonal partial least squares discriminant analysis and clustering analysis to discriminate the parts of *H. cordata* by differentiating the volatile metabolites. It is noteworthy that the pharmacological activities of *H. cordata* are mainly from volatile compounds and flavonoids. Therefore, besides volatile components, the flavonoids are the crucial markers to assess the quality of *H. cordata*. Our study then focused on quantifying five major flavonoids for further application using fresh leaves and powder products in the cosmetic and pharmaceutical industries.

Moreover, the HCA of four *H. cordata* commercial samples also showed that the results were highly similar, proving that these products are pure and unadulterated. Figure S2 shows that the four samples were evidently classified into two clusters (P01 and P04 vs. P02 and P03), and there were no significant differences in drying method that affected the flavonoid contents. The samples P02, P03, and P04 were processed by the freeze-drying method, while P01 was processed by the hot air-drying method. However, a previous study reported that freeze-drying method can retain the quality of herbal compounds [31]. This difference in our result may result from quality of raw herbal materials during farming, harvesting, and storage before processing. Therefore, assessing the quality of *H. cordata* raw materials is essential in research and industrial manufacturing.

## 4. Materials and Methods

### 4.1. Materials and Reagents

Ethanol for the extraction and the HPLC solvents (acetonitrile, glacial acetic acid, formic acid, and methanol) were purchased from Merck KGaA (Darmstadt, Germany). Five chemical standards, including rutin hydrate, hyperin, isoquercitrin, quercitrin hydrate, and quercetin, were provided by Sigma-Aldrich (St. Louis, MO, USA).

### 4.2. Instrumentations

Shimadzu LC-2030C 3D HPLC System (Shimadzu, Tokyo, Japan), Ultrasonic Water Baths (Grant, Cambridge, UK), Memmert Waterbath WNB 14 (Memmert, Bayern, Germany), Hettich EBA20S Portable Centrifuge (Hettich, Tuttlingen, Germany), HPLC Column Phenomenex C18 Prodigy™ ODS-3 100 Å (150 mm × 4.6 mm, 5 µm) (Phenomenex, CA, USA), and HPLC Column Phenomenex C18 Prodigy™ ODS-3 100 Å (250 mm × 4.6 mm, 5 µm) (Phenomenex, CA, USA).

### 4.3. Sampling H. cordata Leaves

A total of 32 samples of *H. cordata* leaves were collected from 32 different provinces of Vietnam from January to March 2022 and authenticated by Dr. Hoang Viet, Department of Ecology-Evolutionary Biology, University of Sciences, Vietnam National University, Ho Chi Minh City. Sample collection information is presented in Table S1. Fresh *H. cordata* leaves were dried at 50 °C (moisture content less than 10%), then ground and sieved through a 0.63 mm sieve to obtain *H. cordata* leaves powder. The powder was stored at room temperature and protected from sunlight. Our research also used four samples of *H. cordata* powder commercially available and circulating in the market (Table S2).

### 4.4. Preparation of Flavonoids Standard Solution

The five standards, including rutin hydrate, hyperin, isoquercitrin, quercitrin hydrate, and quercetin, were dissolved in a 5 mL volumetric flask with methanol to obtain a stock solution of 100.0, 200.0, 100.0, 360.0, and 2.0 µg/mL, respectively. Accurately diluting 1.0 mL of each standard solution into a 5 mL volumetric flask by methanol to obtain 20.0 µg/mL rutin, 40.0 µg/mL hyperin, 20.0 µg/mL isoquercitrin, 72.0 µg/mL quercitrin, and 0.4 µg/mL quercetin. The solutions were then filtered through a 0.45 µm filter before HPLC analysis.

### 4.5. Preparation of Houttuynia Cordata Leaves Sample Solution

Extraction of *H. cordata* leaves powder with optimized conditions has been previously published [32]. Briefly, 0.5 g of *H. cordata* powder was weighed and extracted with 30 mL of 80% ethanol (solid:liquid ratio 1:60, g/L) at 60 °C for 38 min using ultrasound (Ultrasonic bath XUB10), power 75%. The extract was then filtered through Newstar 102 filter paper and dried. The residue was dissolved in 10 mL of HPLC grade methanol, then centrifuged at 3000 rpm for 15 min. The solution after centrifugation was filtered through a filter of 0.45 μm before HPLC analysis.

### 4.6. RP-HPLC Method Optimization for the Analysis of the Flavonoids

The Shimadzu HPLC system model LC-2030C 3D with a 200–800 nm PDA probe (Shimadzu, Japan) was used to optimize the analysis method for flavonoids of *H. cordata* leaves. The acetic or formic acid in water (A) and acetonitrile (B) mobile phases, flow rates of 0.6, 0.9, and 1.0 mL min^−1^, and gradient programs were designed under different conditions and analyzed via Phenomenex C18 columns (4.6 mm, 5 μm) with the length of 250 mm and 150 mm. The sample injection volume was fixed at 2 μL. A resolution value greater than or equal to 1.5, a tailing factor ranging from 1.8 to 2.0, and a number of theoretical plates greater than 5000 were considered ideal for HPLC analysis.

### 4.7. RP-HPLC Method Validation

The optimized HPLC method was validated according to ICH Quality Guidelines Q2(R1) Validation of Analytical Procedures (2018) with parameters including specificity, stability, accuracy, precision, linearity, range, the limit of detection (LOD), the limit of quantification (LOQ), and robustness [33].

#### 4.7.1. Specificity

The specificity of the RP-HPLC method was evaluated by injecting the blank, the standard, and the *H. cordata* extract solution three times with a volume of 2.0 μL to observe interference peaks and compare the retention times and shapes of the peaks.

#### 4.7.2. Stability

The stability of the extracts was evaluated at 0, 3, 6, 9, and 12 consecutive hours with the optimized HPLC method.

#### 4.7.3. Accuracy

The accuracy of the RP-HPLC method was verified by preparing five standard solutions mixed in methanol (HPLC grade), accurately adding approximately 80%, 100%, and 120% of the standards to the test sample. Five standards were analyzed with the optimized HPLC analytical method. Each injection sample was repeated three times. A recovery rate (%) between 98–102% is considered appropriate.

$$\mathrm{Recovery}\;\mathrm{rate}\;(\%)=\frac{(\mathrm{Found}\;\mathrm{amount}\;-\mathrm{Known}\;\mathrm{amount})}{\mathrm{Added}\;\mathrm{amount}}\;\times \;100\%$$

#### 4.7.4. Precision

The repeatability of the RP-HPLC method was performed by analyzing *H. cordata* leaves samples six times. The percentage relative standard deviation (%RSD) was calculated based on the peak areas.

Intermediate precision was validated by analyzing *H. cordata* leaves samples six times over two consecutive days and calculating %RSD.

#### 4.7.5. Linearity and Range

Five standards were diluted to obtain six different concentrations of rutin (5, 10, 25, 50, 100, and 200 μg/mL), hyperin (16, 24, 32, 40, 80, and 200 μg/mL), isoquercitrin (1, 5, 10, 25, 50, and 100 μg/mL), quercitrin (48, 96, 120, 240, 600, and 1000 μg/mL), and quercetin (0.2, 0.5, 1, 2, 4, and 8 μg/mL). Each concentration was injected in triplicate with the same analytical conditions. A calibration curve was constructed using a linear regression equation between mean peak area (x) and standard concentration (y). The correlation coefficient (R^2^) greater than or equal to 0.999 proves the linearity of the analytical method.

#### 4.7.6. LOD and LOQ

The analytical procedure’s limit of detection (LOD) and limit of quantification (LOQ) were determined based on linearity. LOD and LOQ were calculated according to the formula:

$$\mathrm{LOD}=\frac{3.3\;\times \;S}{a};\;\mathrm{LOD}=\frac{10\;\times \;S}{a}$$

in which, S is the standard deviation; a is the slope of the calibration curve y = ax + b.

### 4.8. Quantitative Analysis of Flavonoids Content

The flavonoids of *H. cordata* were quantified using the calibration curve method. The flavonoid contents in the leaves of *H. cordata* were determined by the formula:

$$C=\frac{y\;\times \;V}{\;w\;\times \;(100\%\;-\;M)}\times {\;10}^{-3}$$

in which, C: flavonoid content in 1 g *H. cordata* powder (mg/g extract); y: flavonoid concentration calculated from calibration curve (µg/mL); V: volume of extract (mL); w: weight of *H. cordata* powder (g); and M: moisture content of *H. cordata* powder (%).

### 4.9. Data Analysis

The raw HPLC chromatographic data were examined and exported as *.txt format files using LabSolutions software (Version 5.87 SP1; Shimadzu, Tokyo, Japan). Quantitative data and HPLC method validation were analyzed using Microsoft Excel 2016. HPLC-based flavonoid profiling chromatogram, similarity analysis, principal component analysis (PCA), and hierarchical cluster analysis (HCA) were performed using the software Origin 2021 (Version 9.8; OriginLab Corp., Northampton, MA, USA). All experiments were repeated in triplicate, and results were presented as mean ± SD.

## 5. Conclusions

This study established an effective RP-HPLC method followed by the multivariate analysis to quantify and assess the quality of 32 *H. cordata* leaves samples from different provinces throughout Vietnam and four powder products. From the PCA analysis, two flavonoids, hyperin and quercetin, can demonstrate the quality of *H. cordata* samples from fresh leaves and powder products. For further studies, we will consider various environmental factors, including climate and soil factors, to investigate their effects on the quality of *H. cordata* in particular and medicinal plants in general.

## Figures and Tables

**Table 1:** Different RP-HPLC analysis conditions of *Houttuynia cordata* leave extracts and the results of parameters including retention time, resolution, tailing factor, and the number of theoretical plates.

Column	Elution Program	Flow Rate (mL min^−1^)	Column Temp.	Mobile Phase	Peak	Retention Time (min)	Relative Retention Time (min)	Resolution	Tailing Factor	The Number of Theoretical Plates
Phenomenex 250 mm × 4.6 mm, 5 μm	Program A: 0–15 min: 20% B; 15–25 min: 20–21% B; 25–40 min: 21–70% B.	1	25 °C	AA 0.2% (A):ACN (B)	1	8.07	0.923	―	0.999	4813
					2	9.75	0.892	3.652	―	7394
					3	10.12	0.885	0.457	―	1243
					4	15.62	0.857	6.060	1.126	8756
					5	33.06	0.860	36.027	1.879	193,035
				FA 0.1% (A):ACN (B)	1	8.24	0.943	―	1.016	4864
					2	9.95	0.910	3.697	―	7746
					3	10.33	0.904	0.555	―	2010
					4	16.04	0.880	7.162	1.157	9116
					5	33.23	0.864	33.483	1.961	140,203
	Program B: 0–10 min: 20% B; 10–30 min: 20–21% B; 30–50 min: 21–70% B.	0.9	25 °C	AA 0.2% (A):ACN (B)	1	8.98	1.027	―	0.997	5254
					2	10.84	0.992	3.809	―	8156
					3	11.25	0.984	0.600	―	2513
					4	17.31	0.950	7.688	1.124	10377
					5	39.03	1.015	40.597	1.297	160,129
				FA 0.1% (A):ACN (B)	1	9.01	1.031	―	1.026	5196
					2	10.92	0.999	3.886	―	8315
					3	11.34	0.992	0.645	―	3035
					4	17.54	0.963	8.232	1.156	10489
					5	39.16	1.018	44.222	1.069	287,119
Phenomenex 150 mm × 4.6 mm, 5 μm	Program C: 0–5 min: 20% B; 5–25 min: 20–21% B; 25–45 min: 21–50% B.	0.6	25 °C	FA 0.1% (A):ACN (B)	1	7.88	0.902	―	0.992	7465
					2	9.93	0.909	5.221	―	8870
					3	10.35	0.906	0.909	―	6873
					4	16.45	0.903	10.798	0.904	10,870
					5	37.35	0.971	42.743	1.058	185,427
			40 °C	FA 0.1% (A):ACN (B)	1	5.69	0.651	―	1.019	4905
					2	7.10	0.650	4.184	―	6561
					3	7.40	0.647	0.659	―	2850
					4	11.71	0.643	8.329	0.867	9489
					5	33.83	0.880	59.77	0.999	272,802

AA: acetic acid; FA: formic acid. The peaks are denoted from 1 to 5 are of rutin, hyperin, isoquercitrin, quercitrin, and quercetin, respectively. ― indicates that the parameter was not calculated. The injection volume was fixed at 2 μL in all experiments, and the detection wavelength was 355 nm for analysis of the peaks in chromatograms.

**Table 2:** Validation results of precision, intermediate precision, repeatability, and stability for determination of five flavonoids.

Standards	Precision				Intermediate Precision		Repeatability		Stability	
	Intra-Day (*n* = 6)		Inter-Day (*n* = 6)		Intra-Day and Inter-Day (*n* = 12)		(*n* = 5)		0, 3, 6, 9, and 12 h	
	Mean	%RSD	Mean	%RSD	Mean	%RSD	Mean	%RSD	Mean	%RSD
Rutin hydrate	173,008.3	1.76	175,346.8	1.40	174,177.6	1.13	173,715	1.62	175,213.2	1.45
Hyperin	300,690.2	0.38	310,497.3	0.89	305,593.8	1.80	300,476	0.37	305,351.6	1.69
Isoquercitrin	63,249.83	1.26	667,22.17	1.21	64,986	1.27	63,043	1.37	65,089	1.97
Quercitrin	1,267,000	0.25	1,308,277	0.95	1,287,638	1.80	1,267,369	0.27	1,290,176	1.49
Quercetin	32,355.83	1.14	33,940	1.48	33,147.92	1.12	32,245	0.87	32,882	2.76

**Table 3:** Validation result of accuracy of the quantitative method RP-HPLC.

Standards	Added Conc. (µg/mL)	Detected Conc. (µg/mL)	Recovery * (%)
Rutin (1) (73.13 µg/mL)	58.56	131.71	100.03
	73.12	147.23	101.34
	88.04	160.51	99.25
Hyperin (2) (32.06 µg/mL)	25.64	58.14	101.72
	32.06	64.22	100.31
	38.43	71.16	101.74
Isoquercitrin (3) (10.42 µg/mL)	8.32	18.81	100.84
	10.42	20.73	98.94
	12.48	23.12	101.76
Quercitrin (4) (120.25 µg/mL)	96.16	215.93	99.50
	120.20	241.75	101.08
	144.24	265.87	100.96
Quercetin (5) (1.14 µg/mL)	0.92	2.07	101.09
	1.11	2.23	98.20
	1.41	2.53	98.58

* Mean recovery of three standard additions with RSD < 2%, *n* = 3.

**Table 4:** Calibration curves, correlation coefficient, linear range, LOD, and LOQ were obtained using the optimized RP-HPLC analysis condition for the five flavonoids. Calibration curves are in the form of regression equation y = ax + b, where a is the slope of the line, b is the y-intercept, x is the peak area, and y is the flavonoid content.

Standards	Calibration Curve	Correlation Coefficient (R^2^)	Linear Range (μg/mL)	LOD (μg/mL)	LOQ (μg/mL)
Rutin	y = 0.000190444x − 0.477933	0.9999	5–200	1.41	5.26
Hyperin	y = 0.0000918087x − 1.65027	0.9999	16–200	1.42	7.68
Isoquercitrin	y = 0.000180095x + 0.0143035	0.9999	1–100	0.35	1.03
Quercitrin	y = 0.000177146x − 2.8326	0.9999	48–1000	9.13	33.44
Quercetin	y = 0.0000435515x + 0.0374795	0.9999	0.2–8	0.09	0.22

**Table 5:** Contents of the five flavonoids in *Houttuynia cordata* leaves (mg flavonoid/g extract). Values are presented as mean ± SD (*n* = 3).

Sample	Rutin	Hyperin	Isoquercitrin	Quercitrin	Quercetin
HC01	0.8866 ± 0.0059	1.1959 ± 0.0006	0.5344 ± 0.0008	6.8148 ± 0.0322	0.0249 ± 0.0003
HC02	1.5230 ± 0.0104	2.1001 ± 0.0057	0.8719 ± 0.0098	8.6587 ± 0.0266	0.0505 ± 0.0010
HC03	1.6269 ± 0.0061	1.7714 ± 0.0010	0.5941 ± 0.0043	9.4315 ± 0.0011	0.0373 ± 0.0002
HC04	0.4965 ± 0.0001	0.7087 ± 0.0009	0.3938 ± 0.0005	6.0251 ± 0.0066	0.0655 ± 0.0010
HC05	0.0410 ± 0.0013	0.021 ± 0.0004	0.0265 ± 0.0002	0.2572 ± 0.0055	0.0098 ± 0.0001
HC06	0.7678 ± 0.0016	1.1615 ± 0.0005	0.5380 ± 0.0024	5.3637 ± 0.0256	0.0234 ± 0.0002
HC07	0.5144 ± 0.0012	0.6056 ± 0.0004	0.4051 ± 0.0017	4.3838 ± 0.0078	0.0191 ± 0.0003
HC08	0.2340 ± 0.0001	0.2165 ± 0.0001	0.0742 ± 0.0004	0.4037 ± 0.0004	0.0165 ± 0.0001
HC09	0.7595 ± 0.0002	1.6164 ± 0.0001	0.6194 ± 0.0002	7.1828 ± 0.0047	0.1126 ± 0.0001
HC10	1.3060 ± 0.0080	2.2296 ± 0.0085	0.9085 ± 0.0031	10.8441 ± 0.0763	0.0336 ± 0.0000
HC11	0.3922 ± 0.0072	0.5278 ± 0.0032	0.2566 ± 0.0023	4.1260 ± 0.0244	0.0102 ± 0.0002
HC12	1.2201 ± 0.0035	1.3328 ± 0.0003	0.6561 ± 0.0027	8.8097 ± 0.0237	0.0299 ± 0.0003
HC13	0.4605 ± 0.0042	0.4094 ± 0.0015	0.1632 ± 0.0011	3.7457 ± 0.0342	0.0191 ± 0.0005
HC14	1.6428 ± 0.0048	2.0960 ± 0.0086	0.8210 ± 0.0053	13.1371 ± 0.0314	0.0450 ± 0.0004
HC15	0.9696 ± 0.0062	0.8089 ± 0.0046	0.3204 ± 0.0032	5.7219 ± 0.0258	0.0208 ± 0.0004
HC16	1.4710 ± 0.0236	1.8131 ± 0.0154	0.6079 ± 0.0059	9.4754 ± 0.1420	0.0298 ± 0.0004
HC17	0.7509 ± 0.0014	1.0327 ± 0.0013	0.5109 ± 0.0029	4.9715 ± 0.0129	0.0208 ± 0.0003
HC18	1.3927 ± 0.0091	1.3874 ± 0.0043	0.8232 ± 0.0129	6.8171 ± 0.0277	0.0167 ± 0.0010
HC19	1.0374 ± 0.0036	1.7311 ± 0.0119	0.8956 ± 0.0095	6.9409 ± 0.0330	0.0409 ± 0.0004
HC20	0.7412 ± 0.0208	1.6961 ± 0.0009	0.7570 ± 0.0027	4.3134 ± 0.0874	0.1265 ± 0.0012
HC21	0.8791 ± 0.0073	1.6441 ± 0.0015	0.6607 ± 0.0050	7.6156 ± 0.0397	0.0317 ± 0.0033
HC22	0.7867 ± 0.0123	1.1246 ± 0.0141	0.5793 ± 0.0138	5.9696 ± 0.0698	0.0255 ± 0.0012
HC23	1.2055 ± 0.0043	1.6909 ± 0.0001	0.8860 ± 0.0030	8.4155 ± 0.0126	0.0283 ± 0.0003
HC24	1.0392 ± 0.0028	1.7227 ± 0.0049	0.8921 ± 0.005	6.9002 ± 0.0233	0.0420 ± 0.0007
HC25	0.5392 ± 0.0079	0.4673 ± 0.0044	0.2838 ± 0.0049	4.3088 ± 0.0739	0.0181 ± 0.0003
HC26	0.1838 ± 0.0028	0.2007 ± 0.0037	0.0966 ± 0.0013	1.3089 ± 0.0109	0.0061 ± 0.0003
HC27	0.7167 ± 0.0093	0.5610 ± 0.0095	0.2436 ± 0.0030	4.8100 ± 0.0354	0.0313 ± 0.0002
HC28	0.7465 ± 0.0099	0.5954 ± 0.0031	0.2577 ± 0.0016	5.0576 ± 0.0707	0.0365 ± 0.0004
HC29	1.5310 ± 0.0230	0.8166 ± 0.0065	0.9112 ± 0.0097	4.7169 ± 0.4037	0.0242 ± 0.0003
HC30	1.0867 ± 0.0153	1.4220 ± 0.0136	0.8519 ± 0.0167	7.5632 ± 0.0410	0.0298 ± 0.0002
HC31	1.5716 ± 0.0159	2.1276 ± 0.0065	0.9838 ± 0.0156	7.1727 ± 0.0255	0.0291 ± 0.0001
HC32	1.1626 ± 0.0102	1.5755 ± 0.0115	0.6561 ± 0.0114	9.0582 ± 0.1171	0.0257 ± 0.0004
P01	1.5938 ± 0.0032	0.8370 ± 0.0053	0.9447 ± 0.0434	4.5257 ± 0.0672	0.0248 ± 0.0001
P02	1.0091 ± 0.0121	1.3975 ± 0.0111	0.8203 ± 0.0391	7.4174 ± 0.0144	0.0282 ± 0.0000
P03	1.5291 ± 0.0057	2.1070 ± 0.0061	0.9120 ± 0.0070	7.2609 ± 0.0039	0.0289 ± 0.0002
P04	1.0682 ± 0.0116	1.3654 ± 0.0075	0.5134 ± 0.0058	5.4733 ± 0.0364	0.0209 ± 0.0001

**Table 6:** Eigenvalues of the correlation matrix.

Principal Components	Eigenvalue	Percentage of Variance	Cumulative Variance
1	3.47	69.41%	69.41%
2	0.96	19.14%	88.56%
3	0.34	6.74%	95.30%
4	0.15	3.10%	98.39%
5	0.08	1.61%	100.00%

## Data Availability

Not applicable.

## Supplementary Materials

The following supporting information is available online at https://www.mdpi.com/article/10.3390/molecules28176378/s1, Table S1: Information on collecting *H. cordata* leaves from different regions in Vietnam from January to March 2022; Table S2: Samples of *H. cordata* powder available in market; Table S3: Extracted Eigenvectors; Table S4: Average hyperin and quercetin content of each seven groups; Table S5: Similarity of HPLC-based flavonoid profiling chromatograms of *H. cordata* leaves; Figure S1: The score plot presents the distribution of *H. cordata* leaves samples on the same plane as well as the correlation between them and the first and second principal components (by analyzing five flavonoids); Figure S2: Hierarchical cluster analysis of four *H. cordata* commercial products.
