# Supplementary material for: RP-HPLC-Based Flavonoid Profiling Accompanied with Multivariate Analysis: An Efficient Approach for Quality Assessment of Houttuynia cordata Thunb Leaves and Their Commercial Products
Source: Molecules. 2023 Aug 31;28(17):6378. doi: 10.3390/molecules28176378 (PMC10489801; doi:10.3390/molecules28176378)
Supplement: Supplementary file 1 [file molecules-28-06378-s001.zip › molecules-2549562-supplementary.pdf]

Supplementary Material

RP-HPLC-Based Flavonoid Profiling Accompanied with Multivariate Analysis: An Efficient Approach for Quality Assessment of *Houttuynia cordata* Thunb Leaves and Their Commercial Products

Minh Hien Nguyen [1,2,*], Dieu Ly Ha [1,2], Binh Minh Do [1,2], Ngoc Trong Nghia Chau [1,2], Thi Huong Tran [1,2], Nguyen Thien Han Le [1,2] and Minh Tri Le [1,2,3,*]

[1] School of Medicine, Vietnam National University Ho Chi Minh City, Quarter 6, Linh Trung Ward, Thu Duc District, Ho Chi Minh City 700000, Vietnam; hdly@medvnu.edu.vn (D.L.H.); dbminh.d2017@medvnu.edu.vn (B.M.D.); cntnghia.d2017@medvnu.edu.vn (N.T.N.C.); tthuong.duoc2016@medvnu.edu.vn (T.H.T.); lnthan.d2019@medvnu.edu.vn (N.T.H.L.)
[2] Vietnam National University Ho Chi Minh City, Quarter 6, Linh Trung Ward, Thu Duc District, Ho Chi Minh City 700000, Vietnam
[3] Faculty of Pharmacy, University of Medicine and Pharmacy of Ho Chi Minh City, Dinh Tien Hoang Street, Ben Nghe Ward, 1 District, Ho Chi Minh City 700000, Vietnam
* Correspondence: nmhien@medvnu.edu.vn (M.H.N.); lmtri@medvnu.edu.vn or leminhtri@ump.edu.vn (M.T.L.); Tel.: +84373-696-894 (M.H.N)

Supplementary

Table S1 Information on collecting *H. cordata* leaves from different regions in Vietnam from January to March, 2022

Code	Address (Commune, District)	Province	Region	Time collect	Location coordinates
HC01	Loi Binh Nhon, Tan An	Long An	Mekong Delta	Jan 2022	10°32'14.6"N 106°22'30.9"E
HC02	Yen Do, Pleiku	Gia Lai	Central Highlands	Feb 2022	13°58'52.7"N 107°59'34.4"E
HC03	Ka Don, Don Duong	Lam Dong	Central Highlands	Feb 2022	11°43'39.3"N 108°29'58.8"E
HC04	Vinh Quang, Rach Gia	Kien Giang	Mekong Delta	Jan 2022	10°02'34.7"N 105°04'42.1"E
HC05	Dong Tam, Hai Ba Trung	Ha Noi	Red River Delta	Jan 2022	20°59'54.5"N 105°50'45.4"E
HC06	Sub-village 10, Bac Lieu city	Bac Lieu	Mekong Delta	Jan 2022	9°18'13.6"N 105°44'13.8"E
HC07	Dong Cuu, Gia Binh	Bac Ninh	Red River Delta	Feb 2022	21°04'02.6"N 106°09'13.5"E
HC08	Xuan Phu, Hue city	Hue	North Central Coast	Jan 2022	16°27'52.2"N 107°36'00.8"E
HC09	Thoi Hoa, O Mon	Can Tho	Mekong Delta	Feb 2022	10°07'09.0"N 105°37'12.7"E
HC10	Long Tien, Cai Lay	Tien Giang	Mekong Delta	Jan 2022	10°21'07.8"N 106°08'20.4"E
HC11	Vinh Thanh, Lap Vo	Dong Thap	Mekong Delta	Feb 2022	10°19'47.2"N 105°37'31.7"E
HC12	My Thuy, Le Thuy	Quang Binh	North Central Coast	Feb 2022	17°11'54.4"N 106°48'41.8"E
HC13	Nam Trung, Tien Hai	Thai Binh	Mekong Delta	Feb 2022	20°20'00.2"N 106°32'25.6"E
HC14	Cam Hieu, Cam Lo	Quang Tri	North Central Coast	Feb 2022	16°48'17.9"N 107°02'18.6"E
HC15	Tam Ngoc, Tam Ky	Quang Nam	South Central Coast	Feb 2022	15°31'50.9"N 108°29'03.0"E
HC16	Hoa Tho Tay, Cam Le	Da Nang	South Central Coast	Feb 2022	16°00'56.2"N 108°10'39.8"E
HC17	Dak Nia, Gia Nghia	Dak Nong	Central Highlands	Feb 2022	11°57'09.0"N 107°42'28.9"E
HC18	Song Ray, Cam My	Dong Nai	South East	Mar 2022	10°44'49.1"N 107°18'36.0"E
HC19	Khoi 5, Tan Ky	Nghe An	North Central Coast	Mar 2022	19°09'46.6"N 105°18'20.6"E
HC20	Duc Thuan, Hong Linh	Ha Tinh	North Central Coast	Mar 2022	18°31'49.9"N 105°41'24.7"E
HC21	Dien Son, Dien Khanh	Khanh Hoa	South Central Coast	Mar 2022	12°16'21.7"N 109°04'56.7"E
HC22	An Ninh, Chau Thanh	Soc Trang	Mekong Delta	Mar 2022	9°36'37.2"N 105°54'02.3"E
HC23	Phu Thanh, Tuy Hoa	Phu Yen	South Central Coast	Mar 2022	13°01'59.1"N 109°19'21.2"E
HC24	Binh Tan, La Gi	Binh Thuan	South East	Mar 2022	10°66'71.6"N 107°78'01.8"E
HC25	Kien Thanh, Cho Moi	An Giang	Mekong Delta	Mar 2022	10°31'15.6"N 105°24'19.7"E
HC26	Phuoc Tan, Xuyen Moc	Ba Ria-Vung Tau	South East	Mar 2022	10°33'52.7"N 107°23'05.2"E

HC27	Phuoc Huu, Ninh Phuoc	Ninh Thuan	South East	Feb 2022	11°30'33.2"N 108°52'45.4"E
HC28	Truong Chinh, Kon Tum city	Kon Tum	Central Highlands	Mar 2022	14°22'40.6"N 108°00'52.1"E
HC29	Rach Goc, Ngoc Hien	Ca Mau	Mekong Delta	Mar 2022	8°39'15.0"N 105°02'44.9"E
HC30	Chau Binh, Giong Trom	Ben Tre	Mekong Delta	Apr 2022	10°11'28.8"N 106°33'21.5"E
HC31	Song Mai, Bac Giang city	Bac Giang	Northeast	Apr 2022	21°18'30.6"N 106°10'52.0"E
HC32	Huu Bang, Kien Thuy	Hai Phong	Red River Delta	Mar 2022	20°46'18.7"N 106°39'28.4"E

Table S2 Samples of *H. cordata* powder available in market

Code	Brand	Drying method
P01	Goce	Hot air drying
P02	Quang Thanh	Freeze-drying
P03	Behena	Freeze-drying
P04	Dalahouse	Freeze-drying

Table S3 Extracted Eigenvectors

	Coefficients of PC1	Coefficients of PC2
Rutin	0.48052	–0.30623
Hyperin	0.5115	0.07328
Isoquercitrin	0.48967	–0.0542
Quercitrin	0.47659	–0.11178
Quercetin	0.20137	0.94097

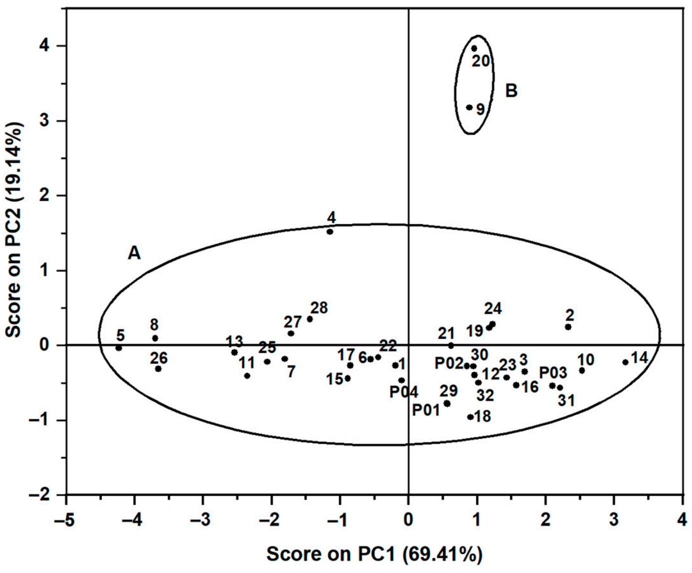

Figure S1 The scores plot presents the distribution of *H. cordata* leaves samples on the same plane as well as the correlation between them and the first and second principal components (by analyzing five flavonoids).

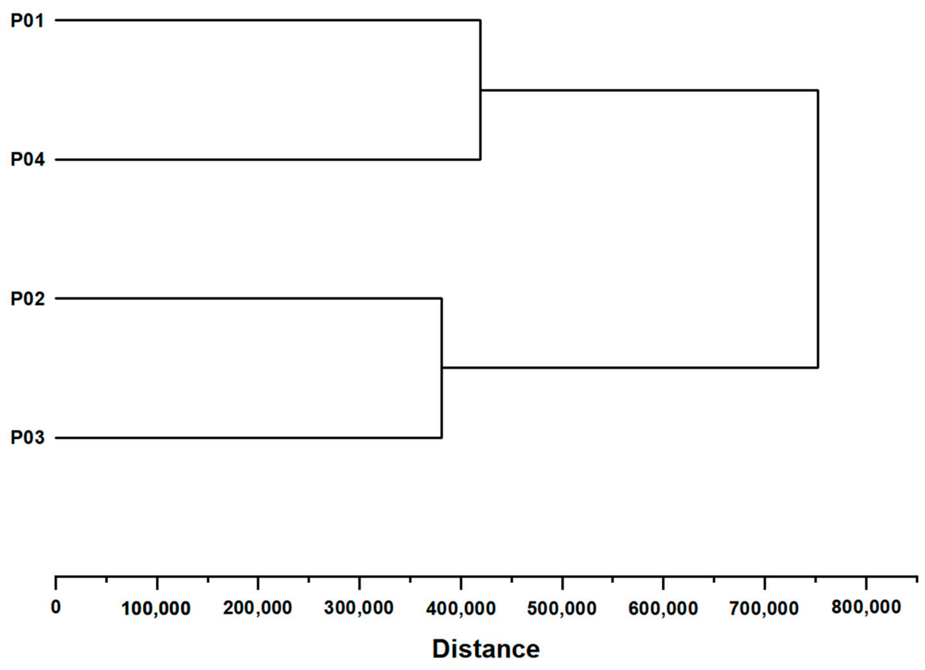

Figure S2 Hierarchical cluster analysis of four *H. cordata* commercial products.

Table S4 Average hyperin and quercetin content of each seven groups.

	Hyperin	Quercetin
Group A	1.26886 ± 0.13372	0.02446 ± 0.00423
Group B	1.70697 ± 0.07343	0.03368 ± 0.00593
Group C	1.65627 ± 0.03986	0.11955 ± 0.00694
Group D	2.13208 ± 0.04998	0.03743 ± 0.00877
Group E	0.55360 ± 0.09061	0.02854 ± 0.01715
Group F	0.82082 ± 0.01183	0.02325 ± 0.00174
Group G	0.14607 ± 0.08867	0.01079 ± 0.00430

Table S5 Similarity of HPLC-based flavonoid profiling chromatograms of *Houttuynia cordata* leaves.

	HC01	HC02	HC03	HC04	HC05	HC06	HC07	HC08	HC09	HC10	HC11	HC12	HC13	HC14	HC15	HC16	HC17	HC18	HC19	HC20	HC21	HC22	HC23	HC24	HC25	HC26	HC27	HC28	HC29	HC30	HC31	HC32	P01	P02	P03	P04
HC01	1.000																																			
HC02	0.992	1.000																																		
HC03	0.999	0.994	1.000																																	
HC04	0.992	0.968	0.986	1.000																																
HC05	0.996	0.982	0.994	0.995	1.000																															
HC06	0.997	0.999	0.997	0.979	0.988	1.000																														
HC07	0.997	0.979	0.993	0.998	0.995	0.988	1.000																													
HC08	0.776	0.847	0.798	0.692	0.749	0.819	0.725	1.000																												
HC09	0.993	0.995	0.992	0.978	0.990	0.997	0.984	0.814	1.000																											
HC10	0.998	0.997	0.997	0.983	0.991	1.000	0.991	0.807	0.998	1.000																										
HC11	0.997	0.978	0.993	0.999	0.996	0.987	1.000	0.722	0.984	0.990	1.000																									
HC12	0.998	0.984	0.997	0.996	0.996	0.991	0.999	0.747	0.986	0.993	0.999	1.000																								
HC13	0.992	0.969	0.989	0.998	0.995	0.978	0.998	0.703	0.975	0.982	0.998	0.997	1.000																							
HC14	0.999	0.987	0.998	0.995	0.997	0.993	0.999	0.758	0.990	0.995	0.999	1.000	0.996	1.000																						
HC15	0.996	0.980	0.996	0.994	0.995	0.986	0.997	0.743	0.980	0.989	0.997	0.999	0.998	0.998	1.000																					
HC16	0.999	0.995	1.000	0.986	0.994	0.998	0.993	0.801	0.994	0.998	0.993	0.996	0.988	0.998	0.994	1.000																				
HC17	0.998	0.998	0.998	0.982	0.990	1.000	0.990	0.810	0.996	1.000	0.989	0.993	0.982	0.995	0.989	0.999	1.000																			
HC18	0.997	0.995	0.998	0.981	0.987	0.997	0.991	0.806	0.989	0.997	0.989	0.994	0.984	0.995	0.992	0.998	0.998	1.000																		
HC19	0.990	0.999	0.990	0.965	0.978	0.998	0.977	0.844	0.996	0.997	0.975	0.981	0.964	0.984	0.974	0.992	0.997	0.992	1.000																	
HC20	0.917	0.956	0.920	0.868	0.900	0.945	0.885	0.925	0.952	0.940	0.884	0.894	0.861	0.904	0.881	0.926	0.938	0.924	0.962	1.000																
HC21	0.996	0.998	0.995	0.979	0.989	1.000	0.987	0.817	0.999	1.000	0.986	0.990	0.978	0.993	0.985	0.997	0.999	0.995	0.998	0.948	1.000															
HC22	1.000	0.994	0.998	0.989	0.993	0.998	0.995	0.786	0.995	0.999	0.994	0.997	0.988	0.998	0.993	0.999	0.999	0.998	0.993	0.926	0.998	1.000														
HC23	0.999	0.996	0.998	0.985	0.991	0.999	0.993	0.799	0.995	1.000	0.992	0.995	0.985	0.996	0.991	0.999	1.000	0.998	0.995	0.933	0.999	1.000	1.000													
HC24	0.990	0.999	0.990	0.965	0.978	0.998	0.977	0.844	0.996	0.997	0.975	0.981	0.964	0.984	0.974	0.992	0.997	0.992	1.000	0.962	0.998	0.993	0.995	1.000												
HC25	0.991	0.967	0.988	0.998	0.992	0.977	0.998	0.694	0.972	0.981	0.998	0.997	1.000	0.995	0.997	0.987	0.981	0.983	0.963	0.857	0.976	0.988	0.984	0.963	1.000											
HC26	1.000	0.990	0.999	0.993	0.996	0.995	0.997	0.771	0.991	0.997	0.997	0.999	0.994	1.000	0.998	0.999	0.997	0.997	0.987	0.910	0.994	0.999	0.998	0.987	0.993	1.000										
HC27	0.992	0.969	0.990	0.997	0.994	0.978	0.997	0.708	0.974	0.982	0.998	0.997	1.000	0.996	0.999	0.988	0.982	0.985	0.964	0.861	0.977	0.988	0.985	0.964	0.999	0.994	1.000									
HC28	0.992	0.969	0.990	0.997	0.994	0.978	0.997	0.708	0.974	0.982	0.998	0.997	1.000	0.996	0.999	0.988	0.982	0.985	0.964	0.861	0.977	0.988	0.985	0.964	0.999	0.994	1.000	1.000								
HC29	0.981	0.968	0.983	0.971	0.967	0.973	0.983	0.746	0.955	0.973	0.980	0.985	0.981	0.982	0.988	0.981	0.977	0.987	0.962	0.858	0.968	0.979	0.979	0.962	0.982	0.983	0.983	0.983	1.000							
HC30	0.999	0.993	0.998	0.989	0.992	0.998	0.996	0.782	0.993	0.999	0.995	0.997	0.989	0.998	0.994	0.998	0.999	0.998	0.992	0.922	0.997	1.000	1.000	0.992	0.989	0.999	0.989	0.989	0.982	1.000						
HC31	0.973	0.994	0.977	0.935	0.955	0.988	0.952	0.895	0.983	0.984	0.950	0.960	0.937	0.964	0.954	0.979	0.985	0.982	0.994	0.976	0.987	0.977	0.982	0.994	0.934	0.969	0.938	0.938	0.948	0.976	1.000					
HC32	1.000	0.992	0.999	0.992	0.996	0.996	0.997	0.775	0.993	0.998	0.997	0.999	0.992	1.000	0.996	0.999	0.998	0.997	0.989	0.916	0.996	0.999	0.999	0.989	0.992	1.000	0.992	0.992	0.981	0.999	0.972	1.000				
P01	0.977	0.967	0.980	0.964	0.961	0.970	0.977	0.757	0.952	0.970	0.974	0.981	0.975	0.978	0.984	0.978	0.975	0.986	0.960	0.859	0.965	0.975	0.976	0.960	0.976	0.979	0.978	0.977	1.000	0.979	0.950	0.977	1.000			
P02	0.999	0.994	0.998	0.989	0.992	0.998	0.996	0.782	0.994	0.999	0.995	0.997	0.988	0.998	0.993	0.998	0.999	0.998	0.993	0.924	0.997	1.000	1.000	0.993	0.988	0.999	0.988	0.988	0.980	1.000	0.977	0.999	0.977	1.000		
P03	0.975	0.995	0.980	0.939	0.959	0.989	0.956	0.891	0.985	0.986	0.953	0.963	0.941	0.968	0.957	0.982	0.987	0.984	0.995	0.975	0.989	0.980	0.984	0.995	0.938	0.972	0.942	0.942	0.950	0.979	1.000	0.975	0.952	0.979	1.000	

P04	0.989	1.000	0.992	0.961	0.977	0.997	0.974	0.861	0.992	0.995	0.973	0.980	0.964	0.984	0.977	0.993	0.996	0.994	0.998	0.959	0.996	0.991	0.994	0.998	0.962	0.987	0.965	0.965	0.967	0.990	0.996	0.989	0.967	0.990	0.997	1.000
